# Beyond checking: verification quality, reliance calibration, and learning in generative AI-assisted higher education

**DOI:** 10.3389/fpsyg.2026.1965371

**Published:** 2026-09-11

**Authors:** Junjie Wei, Yiming Shang

**Affiliations:** 1 School of Intelligent Engineering, Shandong Management University, Jinan, China; 2 School of Civil Engineering, Architecture and Environment, Hubei University of Technology, Wuhan, China

**Keywords:** epistemic cognition, epistemic vigilance, generative artificial intelligence, higher education, learning, reliance calibration, trust, verification

## Abstract

Generative artificial intelligence (GenAI) can support explanation and feedback while also producing fluent claims that are incomplete, unsupported, or false. Higher-education research increasingly examines how students judge, check, and use such outputs, yet adjacent measures can represent different kinds of critical engagement. This Mini Review distinguishes seven analytically ordered measurement targets: epistemic evaluation, verification initiation, process quality, success, reliance decisions, immediate task performance, and independent learning. Reliance calibration is treated separately as an output-contingent classification of reliance decisions. Targeted Web of Science Core Collection searches (2022–2026) yielded 493 unique records; 10 related reviews and 14 priority empirical studies were examined. The searches were targeted rather than systematic. Among the 14 priority studies, none jointly measured verification success and subsequent reliance calibration against an independently adjudicated reference standard; delayed retention or transfer was also uncommon. We identify boundary conditions including prior knowledge, learner characteristics, task stakes, task verifiability, verification costs, accountability, AI system/configuration, and multidimensional AI literacy. The map is an analytic ordering rather than a validated causal model. Educational interventions should therefore be evaluated for the specific process they target, from verification quality to reliance decisions and learning that persists without AI support.

## Introduction

Generative artificial intelligence (GenAI) chatbots are increasingly used in university learning for explanations, feedback, and writing support (Badger et al., 2026). Their educational value carries an epistemic challenge: fluent, authoritative-seeming responses can still be incomplete, unsupported, or false (Hu et al., 2026; Lai et al., 2026). Recent reviews connect GenAI with higher-order cognition, critical thinking, cognitive offloading, learner agency, psychological outcomes, and epistemic autonomy, generally reporting conditional effects (Alubthane, 2026; Balart et al., 2026; Li et al., 2026; Ni and Li, 2026; Suazo et al., 2026; Wang et al., 2026; Zhai et al., 2024). Empirical studies likewise examine how students judge, verify, and use AI-generated information (Choi et al., 2025; Hu et al., 2026; Pudasaini, 2026).

Broad labels such as “critical” or “responsible” AI use can obscure distinct outcomes. A learner may check without using a strong process or resolving uncertainty; reliance measures may not show whether use matched output quality; and a correct final answer does not establish retention, transfer, or unaided performance. These distinctions separate metacognitive monitoring from regulatory action and learning, which is this review’s Educational Psychology focus.

Recent syntheses cover critical thinking, cognitive offloading, learner agency, psychological outcomes, epistemic autonomy, and appropriate reliance (Li et al., 2026; Ni and Li, 2026; Suazo et al., 2026; Wang et al., 2026; Zhai et al., 2024; Raees et al., 2026). The present problem is narrower: What evidence distinguishes a check from successful verification, a reliance decision from reliance calibrated to independently adjudicated AI-output quality, and immediate performance from learning that persists without AI support? This is fundamentally a construct-validity problem: neighboring measures should support distinct interpretations within a nomological network rather than be treated as interchangeable indicators (Cronbach and Meehl, 1955; Messick, 1995).

This Mini Review organizes evidence around seven analytically ordered measurement targets: epistemic evaluation, verification initiation, process quality, and success, reliance decisions, immediate task performance, and independent learning. Reliance calibration is an output-contingent classification of a reliance decision, not an additional chronological target. We conducted 11 complementary topic searches in the Web of Science Core Collection (2022–2026; all editions), updated through 7 August 2026. After Digital Object Identifier, Web of Science accession-number, and title deduplication, 772 exports yielded 493 unique records. Two authors independently screened the corpus and reconciled judgments; 114 review records were identified, 10 closest reviews examined in full text, and 14 priority empirical studies audited in detail. Studies were prioritized when they operationalized a focal target, linked adjacent targets, or exposed a measurement boundary. Full strategies, screening, appraisal, and selection details are provided in Supplementary File 1. The review aims to clarify measurement targets, identify direct linkage gaps, and specify boundary conditions and design priorities.

## From epistemic evaluation to verification action

Epistemic vigilance refers to cognitive and social safeguards used to evaluate communicated information before accepting it as true (Sperber et al., 2010). GenAI complicates these safeguards because linguistic fluency and apparent expertise can be produced without the warrants that similar cues may imply in human communication (Aydeniz, 2026; Hu et al., 2026). Related educational work frames evaluative judgment as the capacity to judge the quality of one’s own and others’ work, including AI outputs (Bearman et al., 2024). Credibility judgments can draw on source cues, plausibility, prior knowledge, and task demands (Hilligoss and Rieh, 2008). In digital information settings, lateral reading offers one strategy for moving beyond the focal text to independent information about sources and corroborating evidence (Breakstone et al., 2021; McGrew, 2024). Empirically, epistemic evaluation can be indexed by credibility judgments, confidence, unsupported-claim detection, source-quality judgments, and stated acceptance or rejection reasons.

Recent university studies document heterogeneous patterns of scrutiny. Choi et al. (2025) found that college students used multiple credibility cues and described different checking practices depending on the academic task and their familiarity with the topic. Hu et al. (2026), studying pre-service science teachers, identified evidence thresholds, verification escalation, and stopping rules: participants decided how far to investigate a claim before using, revising, qualifying, or rejecting it. These findings are consistent with treating trust as situated and adjustable rather than as a stable preference for or against automation (Lee and See, 2004; Hoff and Bashir, 2015).

Awareness of risk is not the same as verification action. In cognitive-audit interviews with 12 highly AI-literate participants, Zainuddin et al. (2026) found selective checking shaped by stakes, domain familiarity, effort, and accountability. Rheu and Cho (2025) reported a nonuniform, dimension-specific pattern: understanding LLM processes was associated with more self-reported fact-checking, whereas some forms of self-efficacy and feature knowledge were associated with stronger machine heuristics and less checking. Amaro et al. (2024) showed that false ChatGPT information can reduce trust, but changed trust does not demonstrate an effective check.

The transition from epistemic evaluation to verification is therefore conditional. Learners may notice reasons for doubt yet decide that checking is unnecessary or costly; unfamiliar or consequential tasks may instead prompt checking despite relatively high trust. Verification should therefore remain analytically separate from AI literacy, skepticism, and awareness of hallucinations.

## Beyond checking: verification quality and success

Verification initiation, process quality, and success answer different questions. Initiation records whether a learner attempted to check a claim. Process quality concerns how checking was conducted, including source independence and competence, claim coverage, and corroboration. Verification success concerns whether checking reached a justified conclusion relative to an appropriate reference standard. For open-ended outputs, success may require graded or multidimensional adjudication; rubrics can score accuracy, completeness, evidential support, and relevance, while expert ratings should report inter-rater reliability and disagreement. Mixed or partially correct outputs may require claim-level or ordinal coding with residual uncertainty reported explicitly. These constructs should not be collapsed, because a strong process can remain inconclusive and a weak process can occasionally reach the correct answer.

Direct evidence for separating action from success comes from designs with known target quality. Zhang et al. (2025) asked 15 students to complete two tasks containing plausible LLM falsehoods while thinking aloud and using external sources. Seven students failed Task 1 despite self-checking or external cross-checking (46.7%; Wilson 95% CI, 24.8%–69.9%). This task-specific estimate should not be generalized as a failure rate; it demonstrates that checking can coexist with unresolved errors.

Other studies expose the same measurement issue from different directions. Chen and Lou (2026) recorded accept/reject decisions for ChatGPT feedback among 78 translation students but lacked independent expert evaluation of each AI suggestion, so acceptance cannot automatically be classified as appropriate or rejection as justified. Urban et al. (2025) exposed students to expert and ChatGPT content containing known correct and incorrect claims, allowing incorporated information to be scored, but did not directly observe checking. The study can therefore connect epistemic characteristics to erroneous uptake without identifying which verification process produced the outcome.

Process quality depends on more than whether checking occurs. Asking the same model to reconsider an answer may reveal inconsistency but does not provide source independence and may reproduce the same unsupported claim. External searching adds independent evidence only when sources are competent, relevant, and not merely derivative. Source independence, source competence, claim coverage, and corroboration are therefore process properties rather than success criteria.

Lateral-reading research illustrates this distinction through movement beyond the focal source, investigation of source competence and provenance, and comparison of independent evidence (Breakstone et al., 2021; McGrew, 2024). In GenAI-assisted learning, measurement should distinguish verification initiation, process quality, and the justified conclusion reached instead of treating checking frequency as sufficient evidence of effective verification.

Future studies can operationalize these distinctions by presenting AI outputs with known or expert-adjudicated quality, logging whether and how learners check them, coding process quality, and scoring whether the check reaches a justified conclusion. Such designs would separate reported vigilance, verification initiation, process quality, and verification success. Table 1 illustrates how representative studies distribute across these measurement levels.

**TABLE 1:** Illustrative empirical studies selected to contrast construct operationalization, AI-system reporting, and study design across verification, reliance calibration, and learning in higher-education GenAI and adjacent AI-advice contexts.

Study/design	AI system/version	Verification: initiation, process quality, success	Reliance: decision and calibration	Task/learning outcome	Diagnostic implication
Urban et al., 2025 Experiment; 98 university students; academic writing with known correct/incorrect claims.	ChatGPT-4o.	Checking not directly observed; accuracy of incorporated claims known; verification process not assessed.	Behavioral uptake of correct/incorrect content; no explicit item-level calibration index.	Writing-product accuracy; no retention or transfer.	Links epistemic factors to erroneous uptake, but does not identify the verification process.
Choi et al., 2025 Qualitative interviews; 25 U.S. college students using GenAI for academic tasks.	Participant-selected GenAI tools (primarily ChatGPT); model/version not standardized.	Credibility judgments and retrospective checking strategies; no objective success criterion.	Not directly measured.	No direct learning outcome.	Credibility assessment and reported checking are upstream processes, not evidence of successful verification.
Chen and Lou, 2026 Mixed methods; 78 translation students; accept/reject logs plus rationales.	ChatGPT-3.5 via OpenAI API (May 2025); standardized prompts.	Follow-up strategies observed; AI-feedback correctness not independently adjudicated.	Direct accept/reject behavior; appropriateness cannot be classified without ground truth.	No independent learning outcome.	Observed reliance decisions should not be equated with calibrated reliance.
Zhang et al., 2025 Think-aloud case study; 15 students; two academic tasks with known plausible falsehoods.	ChatGPT, GPT-3.5 (Oct–Dec 2023).	LLM self-checking and external cross-checking observed; final accuracy scored; 7/15 failed Task 1.	Final use/consolidation observed indirectly.	Immediate task accuracy; no retention or transfer.	Checking can coexist with unresolved error; success requires an outcome criterion/reference standard.
Dávila et al., 2025 Randomized classroom field experiment; final *N* = 59 one-shot and *N* = 82 full four-week period.	Generic “AI” source label (examples: ChatGPT/BARD); no specific model. Advice content was identical to peer condition and 50% correct.	Verification not focal.	Weight on advice and follow/not-follow behavior evaluated against correct vs. incorrect advice; knowledge and gender moderated AI advice-taking.	Immediate quiz performance.	Strong adjacent evidence that reliance can be evaluated against advice quality; not naturalistic open-ended GenAI use.
Zainuddin et al., 2026 Explanatory sequential mixed methods; *N* = 311 scale development, *N* = 241 CFA, 12 cognitive-audit interviews.	General academic GenAI use; no single experimental model/version standardized.	Selective checking described in interviews; no objective verification-success criterion.	Qualitative trust/decision processes; no item-level calibration index.	No direct learning outcome.	Selective checking varied with stakes, familiarity, effort, and accountability.
Hou et al., 2025 Scale development; EFA 800 responses; CFA datasets 730 and 1,173 responses.	Generative AI used freely in course problem-solving activities; model/version not standardized in the scale-validation report.	No verification-success measure.	Self-reported reliance patterns; not linked to correctness of individual AI outputs.	No outcome-based calibration or learning outcome.	Reliance behavior can be measured without showing whether reliance was appropriate to output quality.
Pudasaini, 2026 Cross-sectional SEM; 650 students across four universities.	General GenAI academic-information use; model/version not standardized/reported.	Self-reported verification behavior; no objective success criterion.	Self-reported academic reliance; not conditioned on AI correctness.	No direct learning outcome.	A close credibility–trust–verification–reliance model that still leaves verification success and calibrated reliance unresolved.
Zheng et al., 2025 Field study; 182 students and 315 student–ChatGPT quiz conversations.	ChatGPT-4.	No separate verification measure.	AI competence/relevance, follow/not-follow behavior, and final correctness define reliance patterns; no formal calibration index.	Immediate answer correctness; no delayed learning.	Provides stronger appropriateness-relevant behavioral evidence; even following correct guidance can end in failed application.
Hu et al., 2026 Qualitative interviews plus vignette think-aloud; 20 pre-service science teachers.	Researcher-designed GenAI-style vignettes; not verbatim outputs from any single model.	Evidence standards, verification escalation, and stopping rules elicited; no objective success criterion.	Qualitative use/revise/qualify/reject decisions.	No learning outcome.	Defines upstream truth-assessment and verification processes while deliberately holding model-specific output characteristics constant.

AI system/version is reported when a study standardized the system; “not standardized” indicates participant-selected/general GenAI use or researcher-designed stimuli. Four of the 14 priority empirical studies are not displayed as separate rows: Rheu and Cho (2025), Amaro et al. (2024), Olney and Cade (2025), and Galindo-Domínguez et al. (2026); their roles are documented in Supplementary File 1. EFA, exploratory factor analysis; CFA, confirmatory factor analysis; SEM, structural equation modeling; GenAI, generative artificial intelligence.

## From verification to reliance calibration

After evaluation and any checking, a learner still decides whether to accept, revise, qualify, reject, or defer the AI output. Human-factors research distinguishes trust, a psychological attitude, from reliance, an observable decision to use automation (Lee and See, 2004; Hoff and Bashir, 2015). Educational work likewise models trust, reliance, resistance, and actual use as distinct constructs (Wang et al., 2025), while HCI reviews note heterogeneous definitions of appropriate reliance (Raees et al., 2026). Here, reliance calibration is an item-level, output-contingent classification of whether a reliance decision was appropriate given AI-output quality. For mixed-quality outputs, calibration may be judged at the claim or component level or represented on a graded appropriateness scale, because one response can contain accurate, useful, incomplete, and misleading elements simultaneously. Accepting faulty content can constitute over-reliance, whereas rejecting useful content can constitute under-reliance.

Trust and reliance can diverge. Amaro et al. (2024) showed that false ChatGPT information changed reported trust but did not measure subsequent acceptance or rejection of a specific recommendation. Behavioral reliance can also occur despite expressed reservations. A trust scale therefore cannot substitute for an observed reliance decision and, under the output-contingent definition used here, a reliance decision cannot be classified as calibrated without a reference standard for AI-output quality.

Educational studies operationalize “reliance” in different ways. Hou et al. (2025) developed a large-sample scale of undergraduate reliance during GenAI-supported problem solving, distinguishing reflective, cautious, thoughtless, and collaborative patterns. The scale characterizes reported behavior without tying each pattern to a correct or incorrect AI suggestion; therefore, the scale cannot by itself classify reliance as calibrated. Pudasaini (2026) likewise modeled credibility, trust, self-reported verification, and academic reliance without conditioning reliance on the correctness of the AI information. Such measures are useful for describing use patterns while leaving item-level appropriateness unresolved.

Behavioral designs with a reference standard provide stronger appropriateness-relevant evidence. Dávila et al. (2025) used advice that was correct on 50% of items and found that advice weighting varied with prior knowledge and gender, identifying both as boundary conditions in an adjacent AI-advice setting. Zheng et al. (2025) combined AI competence/relevance, follow/not-follow behavior, and final correctness in 315 student-ChatGPT quiz conversations, distinguishing appropriate and inappropriate reliance and a “failed application” pattern. Appropriate reliance therefore does not guarantee successful task execution.

The key direct-linkage gap is verification success followed by reliance calibration. Among the 14 priority empirical studies, none jointly measured whether verification succeeded and then classified the subsequent reliance decision against an independently adjudicated output-quality standard. Future experiments can manipulate AI correctness, record verification, score process quality and success, and classify the final reliance decision. The ordering is analytic rather than fixed in time: learners may accept then verify, revisit evaluation after post-hoc doubt, or carry calibration outcomes into later encounters.

## From task success to learning

A further distinction separates successful task completion from learning. Here, independent learning is used as an umbrella outcome domain, not a unitary psychological construct. It includes retention, transfer to new problems, unaided task performance, self-regulated use of knowledge, and independent detection of new errors after AI support is withdrawn (Barnett and Ceci, 2002; Dunlosky et al., 2013; Zimmerman, 2002). This framing prevents AI-assisted performance from being treated as evidence that learning persists without assistance. Several studies in the focused evidence set observe an immediate answer, written product, acceptance decision, or quiz score, but such outcomes do not establish retention or transfer. Zheng et al. (2025), for example, could determine whether a final quiz answer was correct but did not include delayed or unaided assessment. Urban et al. (2025) could score the accuracy of content incorporated into academic writing but not whether students later detected similar errors independently. Across Table 1, even stronger behavioral designs generally stop at immediate accuracy rather than delayed, unaided learning.

Correcting or rejecting AI output may become a learning opportunity when it requires substantive cognitive work; simple answer substitution is unlikely to provide the same benefit. Olney and Cade (2025) used an error-correction paradigm in which participants corrected a virtual student and completed pre- and post-tests. Their findings suggest that effort during correction matters for learning. Because the study did not examine naturalistic GenAI use, it provides mechanistic evidence rather than direct evidence about contemporary university GenAI settings. It nevertheless illustrates how correction can support learning when it requires explanation and reconstruction.

Recent syntheses of GenAI, cognitive offloading, learner agency, and higher-order cognition likewise report mixed or conditional relations with critical thinking, autonomy, and cognitive performance (Alubthane, 2026; Ni and Li, 2026; Suazo et al., 2026; Wang et al., 2026; Zhai et al., 2024). A learner may verify a claim and make a defensible reliance decision yet learn little if the cognitive work remains externalized; assistance is more educationally useful when it supports explanation, comparison, and transfer.

**FIGURE 1:**
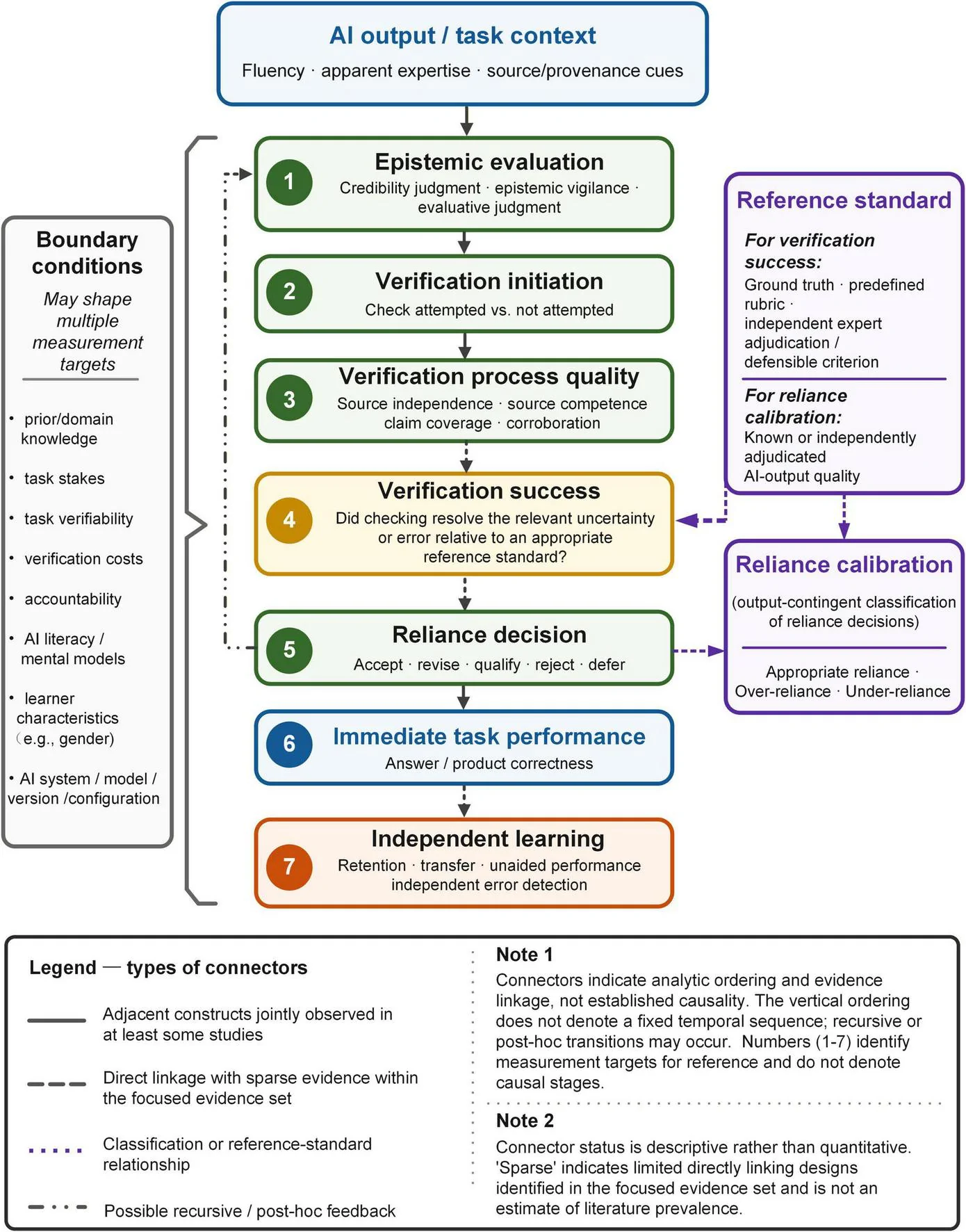
Analytic evidence map for GenAI-assisted higher-education learning. Boxes represent analytically ordered measurement targets or outcomes, not a validated causal sequence. Solid connectors indicate adjacent constructs jointly observed in at least some studies; dashed connectors indicate direct links with limited evidence in the focused set; dotted connectors indicate classification or reference-standard relationships. Verification-process quality concerns how checking is conducted, whereas verification success concerns the justified conclusion reached relative to an appropriate standard. Reliance calibration classifies a reliance decision relative to AI-output quality. Boundary conditions may affect multiple targets. A feedback connector indicates that *post hoc* doubt or prior reliance experience may trigger renewed epistemic evaluation; the ordering is therefore analytic rather than a fixed temporal sequence.

Figure 1 therefore separates immediate task performance from the umbrella domain of independent learning. Stronger evidence would combine process traces with delayed retention, transfer, unaided performance, self-regulated application, or independent error detection. The educational aim is selective AI use that preserves and extends learners’ capacity to judge and reason independently.

## Discussion and research agenda

The proposed distinctions are unlikely to operate uniformly across learners, tasks, or AI systems. Prior/domain knowledge can shape epistemic evaluation, verification success, and, later, reliance calibration; Dávila et al. (2025) also identified gender as a moderator of advice weighting, while Chen and Lou (2026) and Hu et al. (2026) showed that proficiency, unfamiliarity, and uncertainty altered evaluation or verification demands. These findings motivate explicit treatment of learner characteristics rather than assuming homogeneous effects.

Task stakes, verification costs, accountability, and task verifiability enter at different measurement targets. Stakes, costs, and accountability may primarily affect verification initiation and checking depth (Zainuddin et al., 2026; Hu et al., 2026), whereas verifiability constrains whether success can be judged against stable evidence. These mappings are analytic expectations rather than established moderator effects.

Artificial intelligence literacy is multidimensional and may influence evaluation, checking strategy, and reliance decisions (Rheu and Cho, 2025; Zainuddin et al., 2026). AI system, model/version, prompt/configuration, and access date are also boundary conditions because output quality and the applicable reference standard depend on the specific system state. Future studies should report these details explicitly.

The same separation can improve intervention research. A lateral-reading lesson should be evaluated for effects on verification strategy and, separately, verification success; a confidence prompt may primarily alter metacognitive monitoring; a forced-delay interface may change reliance decisions; and an explanation or error-correction activity is more likely to influence learning when it induces substantive processing. Evaluating every intervention with a single self-report measure of “responsible use” would obscure these different mechanisms.

Research designs can make the linkage testable by specifying or adjudicating AI-output quality, capturing verification initiation, coding process quality, scoring verification success, recording the accept-revise-reject decision, and evaluating that decision against AI quality. When learning is intended, immediate performance should be followed by unaided retention or transfer. Supplementary File 1 provides operational definitions, calibration metrics, mixed-effects analysis guidance, multiplicity and reliability recommendations, an illustrative sample-size calculation, methodological appraisal, and design-appropriate reporting standards.

The focused approach limits inference. Searches were targeted rather than systematic, and the evidence set mixes small qualitative studies, direct higher-education GenAI studies, adjacent AI-advice/HCI designs, and foundational or mechanistic sources. The map therefore organizes construct distinctions and missing direct tests; it does not estimate prevalence, effect sizes, or causal sequence. Given the 7 August 2026 search cut-off and rapid model evolution, it should be interpreted as a time-bounded snapshot.

This agenda is more specific than a general call for “critical AI literacy.” Interventions may increase vigilance, improve verification quality or success, alter reliance calibration, or support learning from error correction. Interventions that reduce inappropriate acceptance should also be tested for unintended rejection of correct assistance (Lee and See, 2004; Buçinca et al., 2021). The educational objective is effective, proportionate verification that supports calibrated reliance while preserving the cognitive work required for learning.

## Conclusion

Generative artificial intelligence-assisted higher education requires more than awareness that AI can be wrong. Current studies assess distinguishable measurement targets: epistemic evaluation, verification initiation, process quality, and success, reliance decisions, immediate task performance, and independent learning. Reliance calibration classifies reliance decisions against AI quality. Evidence for individual targets is growing, but their links require validation across disciplines, task types, student populations, and AI systems. Keeping these targets separate provides a clearer basis for research and instruction aimed at effective verification, calibrated reliance, and reasoning that persists without AI support.
