# Supplementary material for: Beyond checking: verification quality, reliance calibration, and learning in generative AI-assisted higher education
Source: Front Psychol. 2026 Sep 11;17:1965371. doi: 10.3389/fpsyg.2026.1965371 (PMC13613975; doi:10.3389/fpsyg.2026.1965371)
Supplement: Supplementary file 1 [file Table_1.docx]

Supplementary File S1

# S1. Purpose and scope

This supplement documents the targeted evidence-identification process used to support the focused Mini Review. The searches were designed to identify evidence relevant to construct definition, measurement separation, and direct linkages among epistemic evaluation, verification, reliance, calibration, and learning in generative-AI-assisted higher education. The process was designed for a focused Mini Review and was not intended to constitute exhaustive or PRISMA-based study selection. The expanded audit below makes the search, screening, prioritization, and interpretation process explicit while preserving that focused-review design.

# S2. Information source and search settings

| **Setting** | **Value** |
| --- | --- |
| Database | Web of Science Core Collection |
| Search field | Topic |
| Publication years | 2022–2026 |
| Editions | All |
| Final documented search date | 7 August 2026 |
| Number of complementary search sets | 11 |

For the two review-collision searches (S4A and S4B), the initial Topic-search results were subsequently restricted to the Web of Science document type “Review Article.” Only the Review Article-filtered records (29 for S4A and 109 for S4B) were exported and included in the 772-record export total.

# S3. Search-set summary and roles

Search-set labels (e.g., S1A, S1B, S2A-Core) are internal organizational identifiers rather than database-generated codes. “Core” indicates a narrowly specified search, whereas “Broad” indicates a more inclusive supplementary search. S4A and S4B were two of the 11 exported search sets, not additional searches outside the 11-set total.

| **Search set** | **Purpose** | **Records** |
| --- | --- | --- |
| S1A | Verification / fact-checking | 157 |
| S1B | Credibility / truth assessment | 14 |
| S2A-Core | Appropriate / calibrated reliance | 2 |
| S2B-Core | Behavioral over-reliance | 11 |
| S3A1 | Epistemic vigilance / trust / authority | 15 |
| S3A2 | Trust calibration | 14 |
| S3B | Trust × accuracy / error / credibility | 35 |
| S4A | Close review collision search | 500 initial; 29 Review Articles exported |
| S4B | Broad review collision search | 1,885 initial; 109 Review Articles exported |
| S2A-Broad | Broad reliance / automation bias | 183 |
| S2B-Broad | Broad behavioral reliance / over-reliance | 203 |

Across the 11 exported search files, 772 records were retained before cross-set deduplication. Because the search sets were deliberately overlapping, set-level counts are not mutually exclusive evidence pools.

# S4. Full Web of Science search strategies

**S1A — Verification / Fact-checking**

*Result: 157 records*

("generative AI" OR "generative artificial intelligence" OR ChatGPT OR "large language model*")
AND
("university student*" OR "college student*" OR undergraduate* OR postgraduate* OR "graduate student*" OR (student* NEAR/5 "higher education"))
AND
(verif* OR fact-check* OR "fact check*" OR "source check*" OR "cross-check*" OR "cross check*" OR "lateral reading")

**S1B — Credibility / Truth Assessment**

*Result: 14 records*

("generative AI" OR "generative artificial intelligence" OR ChatGPT OR "large language model*")
AND
("university student*" OR "college student*" OR undergraduate* OR postgraduate* OR "graduate student*" OR (student* NEAR/5 "higher education"))
AND
("source credibility" OR "information credibility" OR "credibility assessment" OR "credibility evaluation" OR "credibility judgment*" OR "trustworthiness judgment*" OR "truth assessment" OR "information evaluation")

**S2A-Core — Appropriate / Calibrated Reliance**

*Result: 2 records*

("generative AI" OR "generative artificial intelligence" OR ChatGPT OR "large language model*")
AND
("university student*" OR "college student*" OR undergraduate* OR postgraduate* OR "graduate student*" OR (student* NEAR/5 "higher education"))
AND
("appropriate reliance" OR "calibrated reliance" OR "reliance calibration" OR "selective reliance")

**S2B-Core — Behavioral Over-reliance**

*Result: 11 records*

("generative AI" OR "generative artificial intelligence" OR ChatGPT OR "large language model*")
AND
("university student*" OR "college student*" OR undergraduate* OR postgraduate* OR "graduate student*" OR (student* NEAR/5 "higher education"))
AND
(
 (
 ("automation bias" OR overreliance OR "over-reliance")
 NEAR/10
 (accept* OR follow* OR adopt* OR defer* OR decision*)
 )
 OR
 (
 ((accept* OR follow* OR adopt*)
 NEAR/5
 (incorrect OR erroneous OR inaccurate OR misleading OR flawed))
 AND
 (answer* OR response* OR output* OR advice OR recommendation*)
 )
)

**S3A1 — Epistemic Vigilance / Trust / Authority**

*Result: 15 records*

("generative AI" OR "generative artificial intelligence" OR ChatGPT OR "large language model*")
AND
("university student*" OR "college student*" OR undergraduate* OR postgraduate* OR "graduate student*" OR (student* NEAR/5 "higher education"))
AND
("epistemic vigilance" OR "epistemic trust" OR "epistemic authority")

**S3A2 — Trust Calibration**

*Result: 14 records*

("generative AI" OR "generative artificial intelligence" OR ChatGPT OR "large language model*")
AND
("university student*" OR "college student*" OR undergraduate* OR postgraduate* OR "graduate student*" OR (student* NEAR/5 "higher education"))
AND
("trust calibration" OR "calibrated trust" OR "trust miscalibration" OR "miscalibrated trust")

**S3B — Trust × Accuracy / Error / Credibility**

*Result: 35 records*

("generative AI" OR "generative artificial intelligence" OR ChatGPT OR "large language model*")
AND
("university student*" OR "college student*" OR undergraduate* OR postgraduate* OR "graduate student*" OR (student* NEAR/5 "higher education"))
AND
(
 (trust OR distrust)
 NEAR/10
 (accurac* OR reliab* OR credib* OR error* OR incorrect* OR hallucinat* OR misinformation OR falsehood*)
)

**S4A — Close Review Collision Search**

*Initial result: 500 records; after Document Types → Review Article: 29 records exported*

("generative AI" OR "generative artificial intelligence" OR ChatGPT OR "large language model*")
AND
("higher education" OR "university student*" OR "college student*" OR undergraduate* OR postgraduate* OR "graduate student*")
AND
(
 verif*
 OR fact-check*
 OR "fact check*"
 OR "epistemic vigilance"
 OR "epistemic trust"
 OR "trust calibration"
 OR "calibrated reliance"
 OR "appropriate reliance"
 OR overreliance
 OR "over-reliance"
 OR "automation bias"
)

*Additional filter: Document Types → Review Article*

**S4B — Broad Review Collision Search**

*Initial result: 1,885 records; after Document Types → Review Article: 109 records exported*

("generative AI" OR "generative artificial intelligence" OR ChatGPT OR "large language model*")
AND
("higher education" OR "university student*" OR "college student*" OR undergraduate* OR postgraduate* OR "graduate student*")
AND
(
 trust
 OR relianc*
 OR credib*
 OR verif*
 OR fact-check*
 OR "fact check*"
 OR hallucinat*
 OR misinformation
 OR falsehood*
 OR "critical thinking"
 OR "cognitive offloading"
 OR "AI literacy"
 OR epistemic
)

*Additional filter: Document Types → Review Article*

**S2A-Broad — Broad Reliance / Automation Bias**

*Result: 183 records*

("generative AI" OR "generative artificial intelligence" OR ChatGPT OR "large language model*")
AND
("university student*" OR "college student*" OR undergraduate* OR postgraduate* OR "graduate student*" OR (student* NEAR/5 "higher education"))
AND
("appropriate reliance" OR "calibrated reliance" OR "reliance calibration" OR overreliance OR "over-reliance" OR underreliance OR "under-reliance" OR "automation bias")

**S2B-Broad — Broad Behavioral Reliance / Over-reliance**

*Result: 203 records*

("generative AI" OR "generative artificial intelligence" OR ChatGPT OR "large language model*")
AND
("university student*" OR "college student*" OR undergraduate* OR postgraduate* OR "graduate student*" OR (student* NEAR/5 "higher education"))
AND
(
 "automation bias"
 OR overreliance
 OR "over-reliance"
 OR
 ((reliance OR "rely on" OR "relied on")
 NEAR/10
 (advice OR recommendation* OR suggestion* OR answer* OR output* OR decision*))
)

# S5. Record management and deduplication

The 11 exported Web of Science files contained 772 records in total. Records were deduplicated in the following order: (1) DOI, (2) Web of Science accession number (UT), and (3) normalized title. This process yielded 493 unique records. Approximately 223 unique records appeared in the core-mechanism search sets and 203 unique records appeared in the broad reliance sensitivity sets; these pools overlap and therefore should not be summed. The two review-collision searches (S4A and S4B) yielded 114 unique review records after cross-set deduplication.

Conference-proceedings evidence was not automatically outside the Web of Science search. The documented audit records show that several proceedings papers used in the synthesis were retrieved by the structured Web of Science searches, including Rheu and Cho (2025), Zhang et al. (2025), Zheng et al. (2025), and Olney and Cade (2025). Their proceedings venue therefore does not imply that they were added outside the documented search process.

# S6. Independent screening and targeted prioritization workflow

The two authors independently screened the 493 deduplicated records against the documented prioritization criteria. Each author made an independent judgment about whether a record warranted close review-collision assessment, detailed empirical audit, or retention as contextual/sensitivity evidence. The two sets of decisions were then compared, and discrepancies were resolved through discussion and consensus before the focused full-text audits were finalized.

The workflow was designed for targeted prioritization rather than exhaustive eligibility screening. Empirical records received priority for detailed audit when they (1) directly operationalized at least one focal measurement target, (2) linked adjacent targets, or (3) exposed a measurement boundary relevant to verification success, reliance calibration, immediate task performance, or independent learning. Records focused mainly on adoption, attitudes, ethics, or broad AI perceptions without directly informing a focal measurement target or linkage were retained as contextual/sensitivity evidence rather than treated as formal exclusions.

The 114 review records constituted a subset of the 493-record deduplicated corpus. Fourteen empirical studies formed a separate priority empirical subset. The remaining 365 records were retained in the contextual/sensitivity corpus and were not subjected to the same detailed illustrative full-text audit. This accounting is descriptive of a focused Mini Review workflow and should not be interpreted as an exhaustive PRISMA-style eligibility flow.

*Figure S1. Targeted evidence-identification and prioritization flow. The diagram documents a focused prioritization workflow rather than an exhaustive PRISMA eligibility process.*

# S7. Review-collision audit

The 114 unique review records from S4A and S4B were screened to assess whether an existing review already used a near-duplicate organizing question. Ten of the closest reviews were examined at full-text level and classified by conceptual overlap: Level 1 = topical overlap; Level 2 = substantial adjacent overlap; Level 3 = near-duplicate organizing question. The audit identified 0 Level-3 reviews, 6 Level-2 reviews, and 4 Level-1 reviews. The resulting decision was to retain the Mini Review while framing its contribution around construct validity, measurement separation, and under-tested direct linkages rather than around trust, verification, or reliance as topics in themselves.

# S8. Priority empirical full-text audit

Fourteen empirical studies were examined in greater detail because they directly informed one or more focal measurement targets, linkage questions, or boundary conditions. The table below records the role of each study in the focused synthesis. Design and sample descriptors reproduce the documented audit; where the analytic sample varied across analyses or study periods, the table reports that variation rather than forcing a single sample-size value.

| **Study** | **Design / sample** | **Principal role in synthesis** | **Main-text Table 1** |
| --- | --- | --- | --- |
| Urban et al. (2025) | Experiment; 98 university students | Correct/incorrect information integration; epistemic/metacognitive predictors | Yes |
| Choi et al. (2025) | Qualitative interviews; 25 U.S. college students | Epistemic evaluation and reported checking; no objective verification success | Yes |
| Chen and Lou (2026) | Mixed methods; 78 translation students | Accept/reject decisions and follow-up verification; no independent output-quality adjudication | Yes |
| Zhang et al. (2025) | Think-aloud case study; 15 university students | Checking can coexist with unresolved error; known plausible falsehoods | Yes |
| Dávila et al. (2025) | Randomized classroom experiment; analytic N varies by analysis/period | Behavioral advice reliance against advice quality; knowledge/gender boundary conditions | Yes |
| Zainuddin et al. (2026) | Mixed methods; N=311 scale development, N=241 CFA, 12 cognitive-audit interviews | Vigilance–action gap; stakes, familiarity, effort, accountability | Yes |
| Rheu and Cho (2025) | Cross-sectional survey/path analysis; 287 LLM users | AI-literacy dimensions and self-reported fact-checking | No — text-only support |
| Olney and Cade (2025) | Two experiments; undergraduate final analytic N≈92 plus MTurk N=85 | Mechanistic bridge from correction effort to learning | No — mechanistic support |
| Galindo-Domínguez et al. (2026) | Mixed methods; 404 undergraduates | Self-regulation as a boundary condition; self-reported overreliance | No — boundary support |
| Amaro et al. (2024) | Controlled experiment; 62 university students | Trust response to false information; trust ≠ behavioral reliance | No — supporting evidence |
| Hou et al. (2025) | Scale development; EFA 800 responses; CFA datasets 730 and 1,173 responses | Reliance behavior can be measured without output-contingent calibration | Yes |
| Pudasaini (2026) | Cross-sectional SEM; 650 students across four universities | Credibility–trust–verification–reliance associations; no verification success/calibration | Yes |
| Zheng et al. (2025) | Field study; 182 students, 315 student–ChatGPT conversations | Behavioral reliance taxonomy and immediate correctness | Yes |
| Hu et al. (2026) | Qualitative interviews + vignette think-aloud; 20 pre-service science teachers | Evidence standards, verification escalation, stopping rules, action | Yes |

The four priority empirical studies not displayed as separate rows in the main-text Table 1 were Rheu and Cho (2025), Amaro et al. (2024), Olney and Cade (2025), and Galindo-Domínguez et al. (2026). Wang et al. (2025) was cited as related contextual evidence but was not one of the 14 records in the documented priority empirical full-text audit.

# S9. Operational definitions for the focal measurement targets

| **Target** | **Operational definition** | **Example indicators** | **Key safeguard** |
| --- | --- | --- | --- |
| Epistemic evaluation | Judgment of the credibility/quality of AI-generated information before or during use | Credibility judgment; confidence; unsupported-claim detection; source-quality judgment; stated accept/reject reasons | Do not equate skepticism or AI-literacy scores with successful checking |
| Verification initiation | Whether a learner attempted to check a focal claim or output | Check attempted vs not attempted; external search; cross-check; source consultation | Occurrence of checking does not establish process quality or success |
| Verification process quality | How checking was conducted | Source independence; source competence; claim coverage; corroboration; depth | A strong process can remain inconclusive |
| Verification success | Whether checking reached a justified conclusion relative to an appropriate standard | Binary correctness when ground truth exists; graded rubric; expert adjudication; claim-level/ordinal coding | Reference standard must be independent/defensible and uncertainty reported |
| Reliance decision | Observable use decision after evaluation/checking | Accept; revise; qualify; reject; defer; follow/not-follow | Trust attitude is not the same as reliance behavior |
| Reliance calibration | Appropriateness of a reliance decision conditional on AI-output quality | 2×2 correctness × accept/reject classification; graded appropriateness; claim/component-level scoring | Requires a known or independently adjudicated output-quality reference |
| Immediate task performance | Current-task answer/product outcome | Answer correctness; product quality; quiz score | Not equivalent to retention, transfer, or unaided competence |
| Independent learning | Umbrella outcome domain after AI support is reduced/removed | Retention; transfer; unaided performance; self-regulated application; independent error detection | Not a unitary psychological construct |

# S10. Criterion-level MMAT 2018 appraisal of the 14 priority empirical studies

The 14 priority empirical studies were appraised at criterion level using the Mixed Methods Appraisal Tool (MMAT), version 2018 (Hong et al., 2018). The two universal screening questions were applied first, followed by the five criteria for the applicable design category. Ratings are reported as Yes (Y), No (N), or Can't tell (CT). CT was used when the published report did not provide enough information to support a defensible Yes/No judgment; it should not be interpreted automatically as poor study quality. Consistent with MMAT guidance and the heterogeneous purposes of these studies, no single numerical or high/medium/low summary score was calculated, and the appraisal was used to qualify interpretation rather than as an exclusion criterion.

| **Study** | **MMAT design category** | **S1** | **S2** | **C1 / C2 / C3 / C4 / C5** |
| --- | --- | --- | --- | --- |
| Urban et al. (2025) | Quantitative non-randomized | Y | Y | CT / Y / CT / CT / Y |
| Choi et al. (2025) | Qualitative | Y | Y | Y / Y / CT / Y / CT |
| Chen and Lou (2026) | Mixed methods | Y | Y | Y / Y / Y / CT / CT |
| Zhang et al. (2025) | Qualitative | Y | Y | Y / Y / Y / Y / Y |
| Dávila et al. (2025) | Quantitative randomized controlled trial | Y | Y | Y / Y / CT / Y / CT |
| Zainuddin et al. (2026) | Mixed methods | Y | Y | Y / Y / Y / CT / CT |
| Rheu and Cho (2025) | Quantitative descriptive | Y | Y | Y / N / Y / CT / Y |
| Olney and Cade (2025) | Quantitative non-randomized | Y | Y | CT / Y / CT / Y / N |
| Galindo-Domínguez et al. (2026) | Mixed methods | Y | Y | Y / Y / Y / Y / CT |
| Amaro et al. (2024) | Quantitative non-randomized | Y | Y | CT / CT / CT / CT / CT |
| Hou et al. (2025) | Quantitative descriptive / measurement study | Y | Y | Y / CT / Y / CT / Y |
| Pudasaini (2026) | Quantitative non-randomized (cross-sectional analytic) | Y | Y | CT / Y / CT / CT / CT |
| Zheng et al. (2025) | Quantitative descriptive field study | Y | Y | Y / CT / Y / CT / Y |
| Hu et al. (2026) | Qualitative | Y | Y | Y / Y / Y / Y / Y |

*Rating key: Y = Yes; N = No; CT = Can't tell. S1 = Are there clear research questions? S2 = Do the collected data allow the research questions to be addressed? C1–C5 are the five design-specific MMAT criteria defined below.*

| **MMAT category** | **C1–C5 criterion key** |
| --- | --- |
| Qualitative | C1 appropriate qualitative approach; C2 adequate data collection; C3 findings adequately derived from data; C4 interpretation sufficiently substantiated by data; C5 coherence among data sources, collection, analysis, and interpretation. |
| Quantitative randomized controlled trials | C1 randomization appropriately performed; C2 groups comparable at baseline; C3 complete outcome data; C4 outcome assessors blinded where relevant; C5 participants adhered to the assigned intervention. |
| Quantitative non-randomized | C1 participants representative of the target population; C2 measurements appropriate; C3 complete outcome data; C4 confounders accounted for in design/analysis; C5 exposure/intervention administered as intended. |
| Quantitative descriptive | C1 sampling strategy relevant to the research question; C2 sample representative of the target population; C3 measurements appropriate; C4 risk of nonresponse bias low; C5 statistical analysis appropriate. |
| Mixed methods | C1 adequate rationale for mixed methods; C2 components effectively integrated; C3 outputs of integration adequately interpreted; C4 divergences/inconsistencies adequately addressed; C5 each component adheres to the quality criteria of its methodological tradition. |
| **Study** | **Appraisal rationale / reporting limitation** |
| Urban et al. (2025) | The experimental comparison involved 98 students (49 independent-writing; 49 ChatGPT-assisted) and objective integration outcomes. The accessible report did not establish random allocation, so the study was appraised as non-randomized. Representativeness, outcome completeness, and confounder control were not sufficiently documented for a firm Yes rating; measurement appropriateness and delivery of the exposure were supported. |
| Choi et al. (2025) | Semi-structured interviews with 25 U.S. college students and qualitative content analysis were appropriate to the credibility-assessment question. The accessible report supported the approach and data collection, but did not provide enough detail to verify all steps linking coded data to findings or full methodological coherence; those criteria were rated CT rather than inferred. |
| Chen and Lou (2026) | The explanatory sequential design explicitly matched the research questions, with quantitative accept/reject data followed by rationales and interviews and integrated interpretation. Explicit procedures for handling divergence between components and complete component-level quality could not be verified unequivocally, so C4–C5 were rated CT. |
| Zhang et al. (2025) | The qualitative case study used think-aloud tasks and retrospective interviews with 15 university students, documented recruitment and task construction, and described iterative coding and team review. The published methods provided sufficient support for all five qualitative criteria. |
| Dávila et al. (2025) | Students were randomly assigned to AI- versus peer-labeled advice groups in a four-session classroom experiment, and outcomes were behaviorally logged. Analytic sample sizes varied across periods/analyses and some advice was not viewed, so outcome completeness and adherence were rated CT; randomization, baseline comparability, and objective outcome assessment were supported. |
| Zainuddin et al. (2026) | The study used an explanatory sequential design (N=311 scale development; independent CFA N=241; 12 cognitive-audit interviews) with a clear rationale for the qualitative follow-up. Integration was evident, but explicit treatment of divergent results and full component-level appraisal were not sufficiently reported in the accessible record, producing CT ratings for C4–C5. |
| Rheu and Cho (2025) | The survey/path-analysis study sampled 287 LLM users from the United States and Singapore, including college students and young professionals, using established/adapted measures. The sampling strategy was relevant to LLM users but not representative of the higher-education target population; the recruitment denominator was insufficient for assessing nonresponse bias. |
| Olney and Cade (2025) | The repeated-measures experiment counterbalanced condition and topic/test order and used objective pre/post outcomes. Because participants were not assigned to parallel randomized groups, the study was appraised under the MMAT quantitative non-randomized category. Measurement appropriateness and design-based control of order/topic effects were supported. Population representativeness and outcome completeness were not sufficiently documented for firm Yes ratings. Substantial variation in actual correction engagement supported a No rating for intervention/exposure being administered as intended. |
| Galindo-Domínguez et al. (2026) | The study combined validated self-report measures/mediation analyses with qualitative content analysis of open-ended responses from 404 undergraduates. The qualitative findings were used to contextualize the quantitative paradox, supporting integration and handling of divergence; full component-level quality remained CT because of the single-university, self-report quantitative component and limited detail for a complete component-by-component appraisal. |
| Amaro et al. (2024) | The study experimentally exposed 62 university students to correct versus false ChatGPT information and measured trust-related outcomes. Because the accessible report did not provide enough information to verify random allocation, the study was appraised conservatively under the MMAT quantitative non-randomized category. The available information was insufficient to make firm judgments about representativeness, measurement adequacy, outcome completeness, confounder control, or intervention/exposure fidelity; these criteria were therefore rated CT rather than inferred. |
| Hou et al. (2025) | Scale development used EFA on 800 responses and CFA on 730 and 1,173 responses, with appropriate reliability/fit analyses. The sampling process was relevant to scale validation, but population representativeness and the denominator needed to assess nonresponse bias were not sufficiently documented in the accessible report. |
| Pudasaini (2026) | The cross-sectional analytic survey included 650 students from four universities and used covariance-based SEM with measures aligned to the stated constructs. Representativeness, outcome completeness, control of potential confounding, and the exposure criterion were not documented strongly enough for Yes ratings in a non-randomized MMAT frame; the measurement criterion was supported. |
| Zheng et al. (2025) | The field study analyzed 315 student–ChatGPT-4 conversations from 182 students across STEM courses using a four-stage behavioral reliance taxonomy. The sampling strategy and analyses were relevant and measures were behaviorally grounded, while general representativeness and nonresponse/participation bias could not be established from the published report. |
| Hu et al. (2026) | The interpretive qualitative study purposively sampled 20 pre-service science teachers and combined vignette think-aloud with semi-structured interviews. Framework Analysis, iterative deductive/inductive refinement, deviant-case attention, peer debriefing/audit trail, and source-based reconciliation supported all five qualitative criteria. |

*Methodological appraisal reference: Hong, Q. N., Fàbregues, S., Bartlett, G., Boardman, F., Cargo, M., Dagenais, P., et al. (2018). The Mixed Methods Appraisal Tool (MMAT) version 2018 for information professionals and researchers. Education for Information, 34(4), 285–291. doi:10.3233/EFI-180221. The MMAT provides two screening questions and five design-specific criteria for qualitative, randomized, non-randomized, quantitative descriptive, and mixed-methods studies.*

# S11. Main-text Table 1 selection and evidence not tabulated

The 14-study priority empirical audit and the 10-study main-text Table 1 served different purposes. The priority audit examined the measurement and linkage evidence in greater detail, whereas Table 1 was restricted to nonredundant contrasts across epistemic evaluation, verification initiation/process/success, reliance decisions/calibration, immediate task performance, and independent learning. The four priority studies not displayed as separate rows were Rheu and Cho (2025), Amaro et al. (2024), Olney and Cade (2025), and Galindo-Domínguez et al. (2026). These studies remained in the synthesis as supporting, mechanistic, or boundary-condition evidence. The Table 1 selection was therefore not a methodological-quality ranking.

# S12. Recommended measurement, analysis, and reporting specifications for future linkage studies

| **Design issue** | **Recommended specification** |
| --- | --- |
| Quantify the evidence gap | Replace purely qualitative labels where possible with counts from the focused audit. For example, the priority audit identified no study that jointly measured verification success and then classified the subsequent reliance decision against an independently adjudicated output-quality standard. |
| Interval estimate for Zhang et al. (2025) | For Task 1, 7/15 participants ended with an incorrect answer despite engaging with a plausible falsehood: 46.7%, Wilson 95% CI 24.8%–69.9%. This interval should be used only to characterize that small task-specific sample, not literature prevalence. |
| Calibration metrics | When output quality is known, define the 2×2 relation between AI correctness and accept/reject behavior. Depending on the design, report Relative AI Reliance (RAIR) and Relative Self-Reliance (RSR; Schemmer et al., 2022), or equivalent cell-based rates, signal-detection indices such as d′ and criterion c, and—when confidence probabilities are elicited—calibration curves or Brier-type scores rather than mean confidence alone. |
| Analysis model | For item-level calibration data, avoid collapsing all observations to a single participant-level proportion when participants respond to multiple items. Generalized linear mixed-effects models with crossed random intercepts for participant and item are a defensible default when the outcome and design support them; random slopes should be considered when justified. |
| Multiplicity and effect measures | Prespecify one primary endpoint for each principal hypothesis. Treat additional measurement targets as secondary outcomes and control family-wise error or false discovery rate when multiple confirmatory tests are conducted. Report effect estimates with 95% confidence intervals (e.g., risk differences or odds ratios for binary outcomes; standardized mean differences or model-based contrasts for graded outcomes). |
| Rater reliability | For expert-adjudicated outputs, report the number of raters, coding unit, adjudication procedure, and an appropriate reliability statistic: Cohen’s or Fleiss’ κ for nominal coding, weighted κ or Krippendorff’s α for ordinal or multi-rater coding where appropriate, and ICC(2,k), where appropriate, for graded rubric scores. |
| Illustrative sample-size anchor | A worked example should be tied to the prespecified primary endpoint and analysis model. As a simple illustration only, detecting a 10-percentage-point difference in an appropriate-reliance rate (0.60 vs 0.70) with two-sided α=.05 and 80% power requires about 356 independent item-level observations per arm. With 20 items per participant, a design effect of 1+(20−1)×ICC gives approximately 35 participants per arm at ICC=.05 and approximately 52 per arm at ICC=.10. Mixed-model or simulation-based power should replace this approximation for an actual study. |
| Reporting standards | Future trials, observational studies, and qualitative studies should follow the reporting guideline appropriate to their design (e.g., CONSORT and, where applicable, CONSORT-SPI for social, psychological, educational, or behavioural intervention trials; STROBE for observational studies; COREQ or SRQR for qualitative studies; and TIDieR for intervention description) and should report AI model/system, version, access date, full prompts or generation settings where relevant, and any output-sampling or temperature configuration that could affect reproducibility. |

**Methodological reference:** Schemmer, M., Hemmer, P., Kühl, N., Benz, C., and Satzger, G. (2022). Should I follow AI-based advice? Measuring appropriate reliance in human-AI decision-making. Preprint. doi: 10.5445/IR/1000145647.

# S13. Interpretation limits, database coverage, and snapshot date

Terms such as “sparse” and “under-tested” refer specifically to the limited number of directly linking designs identified within this focused evidence set; they are not estimates of prevalence across the entire literature. The evidence map is an analytic ordering of measurement targets and does not establish a causal or fixed temporal sequence. Recursive or post-hoc transitions are plausible and should be tested directly rather than inferred from the ordering.

The structured search used Web of Science Core Collection only. This choice should be interpreted as a reproducible, bounded search rather than a claim of complete database coverage. Proceedings papers that were captured by the documented WoS searches were retained. Sources cited for foundational, contextual, or methodological purposes that were not part of the structured Web of Science corpus were treated separately and were not included in the 493-record denominator.

Because the final documented search date was 7 August 2026 and the GenAI literature is changing rapidly, the evidence map should be interpreted as a snapshot of the literature identified up to that date.

The Web of Science queries did not impose a language filter. All records selected for the 14-study close empirical audit were published in English. Peer-reviewed journal or conference versions were used where available; no preprint was cited in place of an available version of record. The final manuscript uses the published proceedings versions of Zhang et al. (2025) and Zheng et al. (2025). Grey literature was not systematically searched; the structured evidence identification was limited to records indexed in the Web of Science Core Collection.

# S14. Summary of documented evidence-identification outputs

| **Output** | **Count** |
| --- | --- |
| Exported records across 11 search files | 772 |
| Unique records after deduplication | 493 |
| Unique records in core-mechanism search sets | ≈223 |
| Unique records in broad reliance sensitivity sets | ≈203 |
| Unique review records from S4A + S4B | 114 |
| Closest reviews examined at full-text level | 10 |
| Level-3 near-duplicate reviews | 0 |
| Level-2 adjacent reviews | 6 |
| Level-1 topical-overlap reviews | 4 |
| Priority empirical studies examined in greater detail | 14 |
| Illustrative empirical studies included in main-text Table 1 | 10 |
| Priority empirical studies retained as text-only evidence | 4 |
| Remaining contextual/sensitivity records not close-audited | 365 |
